# Insights into the Genetic Connectivity and Climate-Driven Northward Range Expansion of *Turbo sazae* (Gastropoda: Turbinidae) Along the Eastern Coast of Korea

**DOI:** 10.3390/ani15091321

**Published:** 2025-05-02

**Authors:** Young-Ghan Cho, Kyungman Kwon, Hyun Soo Rho, Won-Gi Min, Hee-Do Jeung, Un-Ki Hwang, Yong-Kyun Ryu, Areumi Park, Hyun-Ki Hong, Jong-Seop Shin, Hyun-Sung Yang

**Affiliations:** 1 Tidal Flat Research Center, National Institute of Fisheries Science, 405 Gangbyeonro, Gunsan 54042, Republic of Korea; youngghan@korea.kr (Y.-G.C.); hdjeung83@korea.kr (H.-D.J.); vngi1@korea.kr (U.-K.H.); 2 Tropical & Subtropical Research Center, Korea Institute of Ocean Science and Technology (KIOST), Jeju 63349, Republic of Koreawhdtjqtls@kiost.ac.kr (J.-S.S.); 3 East Sea Environment Research Center, Korea Institute of Ocean Science and Technology (KIOST), Uljin 36315, Republic of Korea; hsrho@kiost.ac.kr (H.S.R.); wgmin@kiost.ac.kr (W.-G.M.); 4 Jeju Bio Research Center, Korea Institute of Ocean Science and Technology (KIOST), Jeju 63349, Republic of Korea; ykyou0111@kiost.ac.kr (Y.-K.R.); areumi1001@kiost.ac.kr (A.P.); 5 Department of Marine Biology and Aquaculture, Gyeongsang National University, Tongyeong 53064, Republic of Korea; hyunki@gnu.ac.kr

**Keywords:** *Turbo sazae*, genetic connectivity, climate-driven range expansion, haplotype diversity

## Abstract

*Turbo sazae*, a commercially and ecologically significant marine gastropod traditionally found in Jeju Island and the southern coast of Korea, is experiencing a reported northward expansion into the East Sea, likely influenced by rising seawater temperatures. This study provides preliminary genetic insights into the genetic structure and connectivity of *T. sazae* populations between Jeju and the East Sea using mitochondrial COI sequences. Samples from 6 geographically distinct locations were analyzed, with three cloned replicates generated to enhance sequence reliability. Genetic diversity, haplotype distribution, and population differentiation were then assessed. Our analysis reveals potential genetic connectivity between Jeju and East Sea populations, possibly driven by larval dispersal via the Kuroshio and Tsushima Currents, highlighted by the predominance of shared haplotype EJ1 (60.0% in Jeju, 50.0% in East Sea). Bayesian phylogenetic analysis estimated the time to the most recent common ancestor (MRCA) between Jeju and East Sea populations at approximately 9.7 to 23.3 million years ago, indicating ancient divergence rather than very recent separation. Pairwise FST values and AMOVA results showed generally low levels of genetic differentiation. Given the small sample sizes and use of a single mitochondrial marker, these findings should be interpreted cautiously as preliminary evidence. Nevertheless, this study highlights the need for continued genetic monitoring of *T. sazae* populations under climate-driven range shifts and provides a foundation for future research incorporating broader genomic approaches.

## Simple Summary

The marine gastropod *Turbo sazae*, commonly harvested in Jeju Island and the southern coast of Korea, has historically been well-established. Recent reports, however, indicate its northward expansion into the East Sea, Korea, is likely driven by increasing ocean temperatures. This study investigates the genetic connectivity among *T. sazae* populations in Jeju and the East Sea to elucidate the mechanisms behind this expansion. Genetic analysis of samples from six locations reveals a high degree of population connectivity that is facilitated by larval transport via ocean currents. A predominant genetic type is shared between both regions, indicating potential contributions from Jeju individuals to newly established populations in the East Sea. These findings offer novel insights into the impacts of climate change on marine species distribution dynamics.

## 1. Introduction

Climate change is causing substantial alterations in the distribution of marine species, leading many taxa to shift poleward in response to increasing ocean temperatures [1,2]. Long-term monitoring indicates a rise of approximately 1.7 °C in sea surface temperatures of the East Sea over the past five decades, with pronounced winter warming [3,4,5]. This warming trend is anticipated to facilitate northward expansions of warm-water species, while cold-adapted species may experience habitat loss or range contraction. For instance, recent analyses in the Barents Sea revealed that numerous benthic invertebrate species, including mollusks and polychaetes, have expanded their ranges northward over the past century, coinciding with rising bottom-water temperatures and reduced sea ice extent [6]. Similarly, fossil records suggest that both warm- and cold-water marine invertebrates faced predictable extinction risks during past global warming events due to shifting thermal habitats and poleward displacement [7].

These shifts carry significant ecological and economic implications, particularly for commercially valuable marine species. Changes in species composition and migration patterns can impact local fisheries, disrupt existing resource management strategies, and necessitate policy adjustments to accommodate new arrivals. In Korea, such trends are evident in species like the top shell *Turbo sazae* Fukuda, 2017, a marine gastropod inhabiting rocky subtidal zones across the Korean Peninsula and Japan. Historically, *T. sazae* was believed to be restricted to Jeju Island and the southern coast of Korea, often misidentified in earlier studies as *Turbo cornutus* ([Lightfoot], 1786) [8]. *T. sazae* has been a crucial fisheries resource in Jeju Island [9,10], harvested alongside abalone by traditional female divers (haenyeo) for generations. However, in recent decades, Jeju’s *T. sazae* populations have sharply declined due to overexploitation and climate-induced habitat changes, including rising seawater temperatures affecting larval settlement success [11]. In contrast to its decline in Jeju, *T. sazae* has recently been documented in the East Sea, historically absent from the area. Market surveys confirm that *T. sazae* harvested from the East Sea is now sold in local seafood markets, indicating successful establishment in these waters. This northward expansion is likely facilitated by the Tsushima and Kuroshio Currents, influencing larval dispersal and genetic connectivity between populations. The warm-water Kuroshio Current originates in the western Pacific, flowing northward along Jeju and the Korean Peninsula before branching into the Tsushima Current, entering the East Sea. If *T. sazae* larvae are transported via these currents, Jeju populations could serve as a genetic source for newly established populations in the East Sea. However, alternative factors such as local adaptation, limited larval exchange, or independent introductions may also contribute to genetic differentiation between populations.

Population genetic studies within the Turbinidae family have traditionally employed mitochondrial genes such as cytochrome c oxidase I (COI) and 16S rRNA due to their high mutation rates and maternal inheritance, while nuclear markers like internal transcribed spacer (ITS), 28S rRNA, and microsatellites provide complementary insights into biparental gene flow and recombination events [12,13,14,15]. In *T. sazae*, Kojima et al. [16] conducted a landmark study examining genetic differentiation among Japanese populations using COI, and Yanagimoto et al. [17] applied COI and 16S rRNA to reveal genetic divergence between the Sea of Japan (East Sea) and Pacific populations.

This study aims to provide important background for future investigations into the genetic structure and northward expansion of *T. sazae* by examining populations from Jeju Island and the East Sea. Specifically, this preliminary study begins to address the following questions: Is genetic evidence supporting the recent establishment of *T. sazae* populations in the East Sea? Do populations from Jeju and the East Sea demonstrate genetic connectivity indicative of larval transport via ocean currents? Alternatively, do these populations exhibit genetic differentiation, suggesting independent lineage formation or adaptation to local conditions? To address these inquiries, we employed COI to assess the genetic diversity and structure among *T. sazae* populations, constructing haplotype networks. This investigation aims to offer early insights into the mechanisms behind species range expansion and their repercussions for fisheries management.

## 2. Materials and Methods

### 2.1. Study Sites and Sample Collection

**Figure 1:**
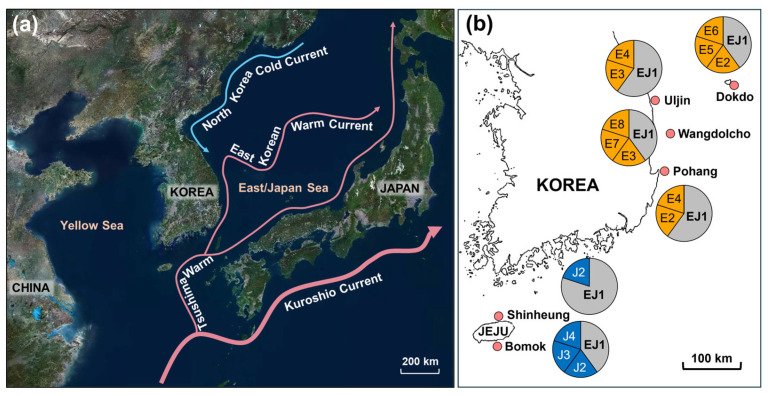
Map of the study area indicating sampling sites and major ocean currents: (**a**) An oceanographic map delineating significant warm and cold currents affecting the Korean Peninsula; (**b**) The locations of sampling sites in Korea, encompassing Jeju and the East Sea. Pie charts represent the proportional haplotype composition at each site, with shared (EJ1) and site-specific haplotypes indicated.

Samples of *T. sazae* with a shell height (i.e., a linear distance from the top to the base of the shell) ranging from 60 to 100 mm, which corresponds to estimated ages of approximately three to six years, were collected from six sites across Jeju Island and the East Sea (Table S1). Site selection was based on documented occurrences of *T. sazae* and to encompass populations from diverse oceanic current systems. Specifically, Jeju Island is primarily influenced by warm currents originating from the Kuroshio Current, including the Jeju warm current, while the East Sea is more directly affected by the Tsushima Current, a major branch of the Kuroshio flowing through the Korea Strait (Figure 1). At each site, 5 individuals were collected via SCUBA diving from rocky shore habitats at depths of 5–10 m.

**Figure 2:**
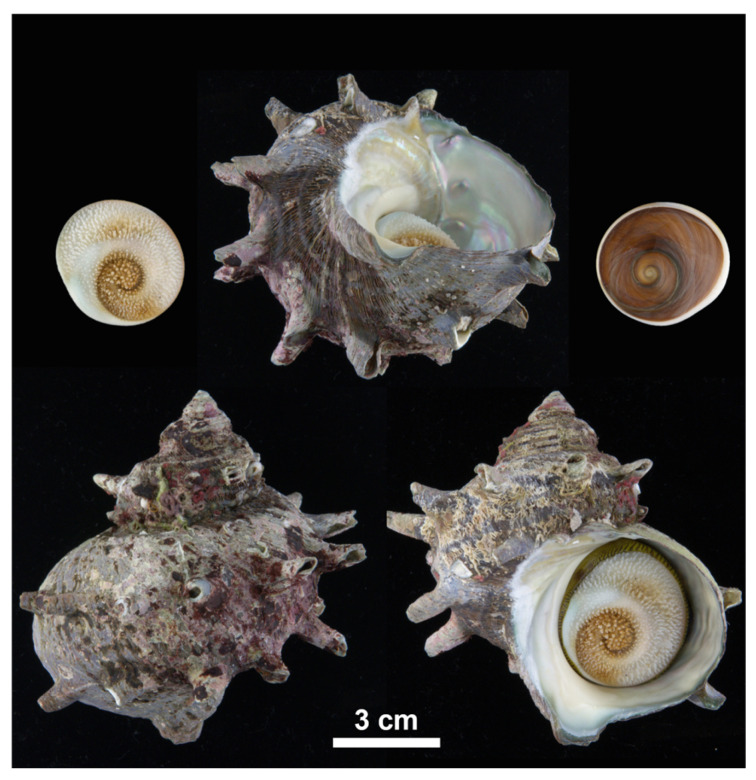
Representative shell morphology of the top shell *Turbo sazae* collected in the present study. Specimens displayed a thick, turban-shaped shell with robust axial spines, a broad aperture with a nacreous interior, and a well-developed operculum. The upper left and right show the calcareous operculum from both inner and outer surfaces.

Upon arrival at the laboratory, specimens were morphologically identified based on established morphological criteria (Figure 2; see also [8] for the diagnostic characteristics distinguishing *T. sazae* from related species). Samples were shucked, and muscle tissues were preserved in 99% ethanol at −80 °C for subsequent analysis.

### 2.2. DNA Extraction, PCR, and Sequencing

Genomic DNA was extracted from 15 mg of soft tissue using the DNeasy Blood and Tissue Kit. For genetic analysis, the mitochondrial COI gene was chosen as the target marker due to its well-established utility in species identification and population genetics of marine gastropods [12]. In addition, the use of this marker also allows direct comparison with published *T. sazae* haplotype networks [17]. A 709-base pair (bp) of the COI region was amplified via polymerase chain reaction (PCR) using a universal primer set (LCO1490 and HCO2198, [18]) under the following PCR conditions: an initial denaturation step at 94 °C for 2 min, followed by 35 cycles consisting of denaturation at 94 °C for 30 s, annealing at 55 °C for 30 s, and extension at 72 °C for 1 min, with a final extension step at 72 °C for 7 min. Positive PCR products were then cloned into the pUC118 vector, and recombinant clones were sequenced using a 3730xl DNA analyzer (Appled Biosystems, Foster City, CA, USA).

### 2.3. Genetic Data Processing and Analysis

The obtained sequences were manually assembled using ApE software v3.1.7 [19], and sequence alignment was conducted using the ClustalW algorithm implemented in MEGA 12 [20]. To evaluate the genetic diversity of *T. sazae* populations, we calculated the number of haplotypes, haplotype diversity (Hd), nucleotide diversity (π), and average nucleotide differences (K) using DnaSP v6.0. Genetic distances were estimated based on the Kimura two-parameter model. Among the 30 COI sequences analyzed, 11 unique haplotypes were deposited in GenBank under accession numbers PV164344–PV164354. Additionally, an analysis of molecular variance (AMOVA) and pairwise genetic differentiation index (F_ST_) were computed using Arlequin 3.5 [21] to evaluate genetic differentiations.

Haplotype networks were visualized using PopART v1.7 [22], employing the median-joining (MJ) network inference method. In addition to the haplotypes identified in this study, previously reported haplotypes associated with the Tsushima Current (CT) and Kuroshio Current (CK) from Yanagimoto et al. [17] were integrated into the analysis to enhance the understanding of genetic connectivity between populations. Since the sequences obtained in the present study (709 bp) were longer than those reported in the previous study (643 bp), we excluded terminal regions to retain only the overlapping 643 bp segment. Sequence trimming was performed manually in MEGA 12.

To estimate the time to the most recent common ancestor (TMRCA) between *T. sazae* populations from Jeju Island and the East Sea, we conducted a Bayesian phylogenetic analysis using BEAST v2.7.1 [23]. The HKY + Gamma substitution model was selected based on Bayesian Information Criterion (BIC) scores calculated in MEGAX. A strict molecular clock was applied, and tip taxa were grouped according to their geographic origin (Jeju or East). To evaluate the divergence time under varying assumptions of molecular evolution, we implemented two substitution rates for COI in marine gastropods as used by previous studies: 1% per million years (0.01 substitutions/site/Myr) [14,24] and 2.4% per million years (0.024 substitutions/site/Myr) [25]. Each BEAST run used a chain length of 10,000,000 generations, with sampling every 1000 steps and a 10% burn-in. Tracer v1.7.2 was used to verify effective sample sizes (ESS > 200) and convergence. Maximum clade credibility (MCC) trees were generated using TreeAnnotator, and the divergence times were visualized in FigTree v1.4.4.

### 2.4. Ethics Statement

This study was approved by the review board of the Korea Institute of Ocean Science and Technology (KIOST) and was conducted in strict compliance with the standard operating guidelines provided by the Animal Experiment Ethics Committee, as stipulated by the Animal and Plant Quarantine Agency of South Korea. Further, all experimental procedures followed bioethical research guidelines as well as international recommendations for the treatment of animals in behavioral research and teaching, according to ASAB/ABS (2018).

## 3. Results

### 3.1. Haplotype Frequency and Distribution

Eleven haplotypes were identified, and their frequency distributions are presented in Table 1. The most prevalent haplotype, EJ1, was found in both Jeju and East Sea populations, representing 60.0% of Jeju individuals and 50.0% of East Sea individuals. Additionally, site-specific haplotypes were observed, particularly in Dokdo (DD), Wangdolcho (WD), and Bomok (BM), suggesting potential localized differentiation within the East Sea populations.

### 3.2. Genetic Diversity Analysis

Genetic diversity metrics, including haplotype diversity (Hd), nucleotide diversity (π), and average nucleotide difference (K), are summarized in Table 2. Haplotype diversity was generally higher in the East Sea populations (mean Hd = 0.747) compared to the Jeju populations (mean Hd = 0.644). Dokdo, Wangdolcho, and Bomok exhibited the highest haplotype diversities (Hd = 0.900), while Shinhung showed the lowest (Hd = 0.400).

In terms of nucleotide diversity, the Jeju populations showed two times higher values compared to the East Sea populations. The highest nucleotide diversity (π = 0.008) was observed in the Bomok (BM) population, followed by Shinhung (π = 0.006). In contrast, the lowest nucleotide diversity (π = 0.001) was found in Uljin (UJ).

The average nucleotide difference (K) followed a similar pattern to nucleotide diversity. The highest K value was observed in Bomok (K = 5.300), followed by Shinheung (K = 3.600), both in Jeju. In contrast, Uljin (K = 0.800) had the lowest K values, indicating minimal nucleotide differences among individuals in these populations.

### 3.3. Genetic Differentiation and Population Structure

**Figure 3:**
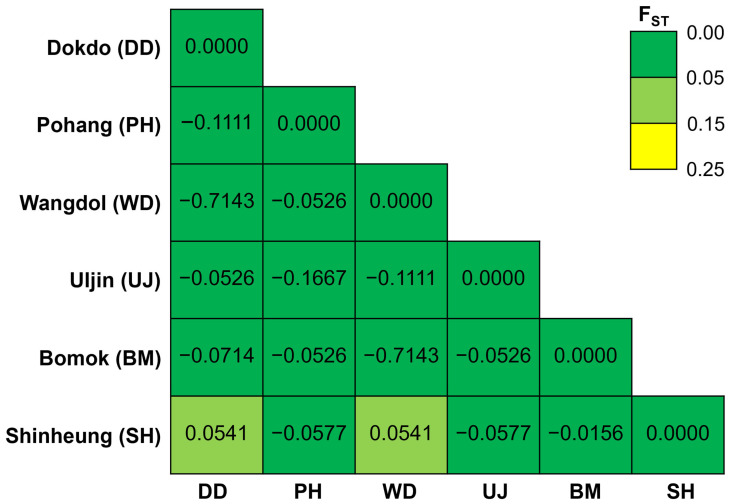
Pairwise F_ST_ values among *Turbo sazae* populations from Jeju Island and the East Sea. The heatmap represents genetic differentiation between populations, with lower F_ST_ values (green) indicating higher genetic connectivity and higher F_ST_ values (yellow) indicating greater genetic differentiation.

The pairwise F_ST_ values revealed minimal genetic differentiation among most populations, with nearly all comparisons exhibiting low or negative F_ST_ values (Figure 3). Notably, the genetic differentiation between Shinheung (SH) and Dokdo (DD) or Shinheung (SH) and Wangdolcho (WD) was the highest among all comparisons (F_ST_ = 0.0541), albeit still low in absolute terms and not statistically significant. Other comparisons, such as between Pohang (PH) and Uljin (UJ) (F_ST_ = –0.1667) and Dokdo (DD) and Wangdolcho (WD) (F_ST_ = –0.7143), yielded negative values, indicating a lack of genetic differentiation.

An analysis of molecular variance (AMOVA) indicated that the vast majority of genetic variation (104.53%) was attributed to the variation within populations, while only 2.44% of the variation occurred between the Jeju and East Sea populations (Table 3). Notably, the genetic variation among populations within regions was negative (−6.97%), indicating negligible or no population structure at this level.

### 3.4. Time to the Most Recent Common Ancestor (MRCA)

The Bayesian phylogenetic analysis revealed that the MRCA of the East Sea and Jeju populations of *T. sazae* was estimated at approximately 9.7 million years ago (Mya) under the 2.4% substitution rate or 23.3 Mya under the 1% substitution rate.

### 3.5. Haplotype Network Analysis

**Figure 4:**
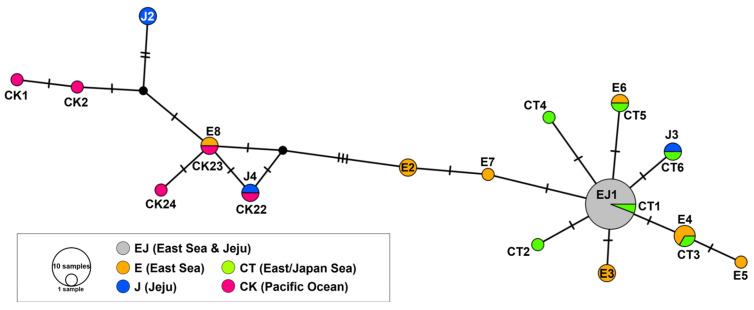
Median-joining haplotype network of *Turbo sazae* based on COI sequences (643 bp). Each circle denotes a unique haplotype, and its size is proportional to the number of individuals sharing that haplotype. Colors represent the geographic origin of each haplotype. The CT and CK haplotypes, which are associated with the Tsushima Current and the Kuroshio Current, respectively, were included from previously published data [17] for comparative purposes.

The median-joining haplotype network displayed a partially star-like topology, with the dominant haplotype EJ1 positioned at the core and connected to several low-frequency variants (Figure 4). EJ1 was the most prevalent haplotype and was shared between both East Sea and Jeju populations, comprising a large proportion of individuals sampled across regions. Surrounding EJ1 were several East Sea-specific haplotypes (E2–E8), each connected by one to three mutational steps, suggesting localized diversification. Among the Jeju-specific haplotypes (J2–J4), J3 was directly connected to EJ1 via a single mutational step, implying close evolutionary proximity. In contrast, J2 and J4 were located further from the network center, connected through longer mutational paths, indicating more distinct evolutionary lineages.

Notably, several haplotypes identified in this study matched previously reported haplotypes associated with the Tsushima Current (CT1–CT6) and Kuroshio Current (CK1–CK24), as referenced in earlier Japanese studies [17]. The network topology showed that CT haplotypes were connected to the central node EJ1, with links to individuals from both the East Sea and Jeju. In contrast, CK haplotypes were located on a more divergent branch, forming a distinct peripheral cluster separated by multiple mutational steps from EJ1.

## 4. Discussion

This study provides preliminary genetic evidence that may support the northward range expansion of T. sazae into the East Sea and highlights the significant role of ocean currents in facilitating population connectivity. Results derived from analyses of haplotype diversity, nucleotide diversity, genetic differentiation (F_ST_), AMOVA, and haplotype network reconstruction indicate a complex population structure characterized by substantial connectivity between populations in Jeju and the East Sea, alongside localized differentiation within specific regions.

### 4.1. Validation of Species Identification and Sequencing Approaches

The accuracy of species identification is a fundamental prerequisite for reliable population genetic studies. In the present study, we emphasize that the specimens used were morphologically consistent with the diagnostic features of *T. sazae*, as described by Fukuda et al. [8]. Although other *Turbo* species occur in nearby waters, no morphologically ambiguous individuals were encountered, and all examined specimens were clearly assignable to *T. sazae*. Subsequent COI sequencing further supported this assignment, as all samples clustered within the known *T. sazae* lineage in phylogenetic analyses, providing dual confirmation via morphology and genetics. In addition, this study employed a triplicate cloning approach for each individual, generating three independent COI sequences per specimen. This strategy was originally implemented to detect potential intra-individual mitochondrial variation, such as heteroplasmy, which has been reported in other mollusks [26,27]. Consequently, one representative haplotype was retained per individual for downstream population genetic inference, indicating there was no heteroplasmy.

### 4.2. Genetic Connectivity

The predominance of the shared haplotype EJ1 in both Jeju and East Sea populations indicates robust genetic connectivity mediated by ocean currents. Additionally, our analyses also revealed that certain Korean samples are genetically identical to haplotypes previously documented in Japanese populations of *T. sazae* (e.g., EJ1 = CT1; E4 = CT3; E6 = CT5; E8 = CK23; J3 = CT6; J4 = CK22) [17]. This finding suggests that *T. sazae* populations in Korea and Japan are not entirely isolated; rather, they share both historical and potentially ongoing genetic exchange, likely facilitated by oceanographic currents such as the Tsushima Warm Current.

Kojima et al. [16] classified *T. sazae* haplotypes into two distinct genetic lineages corresponding to the Kuroshio Current (Pacific lineage) and the Tsushima Current (Japan Sea lineage), suggesting that major ocean currents have historically structured *T. sazae* population. In our study, the detection of Tsushima-associated haplotypes (CT) and a Kuroshio-associated haplotype (CK) provides strong evidence that Korean populations likely originated from both lineages. These results support the hypothesis that historical gene flow between populations in the East China Sea and the Japan Sea has occurred via the Kanmon Strait, a narrow channel separating Kyushu and Honshu. Geological and oceanographic studies suggest that the Kanmon Strait has periodically served as a corridor for marine species migration, particularly during periods of sea level rise and current intensification over the past ~5000 years [16]. During these times, the Tsushima Current, which branches from the Kuroshio Current, could have facilitated the northward transport of larvae and genetic material across regions. Thus, the coexistence of both Kuroshio- and Tsushima-associated haplotypes in Korean *T. sazae* populations implies a complex colonization history shaped by historical dispersal events via ocean currents and subsequent gene flow across oceanographic boundaries.

While our median-joining haplotype network suggests a partially star-like topology centered on haplotype EJ1, its true demographic significance may be further clarified by integrating haplotype frequency information from previous studies. In particular, the median-joining network from Yanagimoto et al. [17] confirms that CT1 (identical to EJ1 in the present study) is the most abundant haplotype in Japanese *T. sazae* populations, reinforcing our interpretation of EJ1 as a potentially ancestral or dominant haplotype across regions. Furthermore, peripheral haplotypes such as CK22, CK23, CK1, and CK2 exhibited considerable frequencies in Japanese populations. This cross-validation strengthens the inference that the observed topology in our study is not an artifact of sampling bias but reflects meaningful regional genetic structuring shaped by both historical connectivity and current-driven larval dispersal.

Furthermore, our Bayesian phylogenetic analysis revealed that the MRCA of the Jeju and East Sea populations was estimated at approximately 9.7 million years ago (assuming a 2.4% substitution rate) or 23.3 million years ago (assuming a 1% substitution rate). This suggests that the genetic divergence between these populations fundamentally dates back to the Miocene, a period characterized by major oceanographic and climatic changes in the northwestern Pacific. The early Miocene to late Miocene epochs witnessed the development of the Tsushima and Kuroshio Current systems [16,17,28]. Thus, while recent dispersal via the Kanmon Strait likely facilitated the secondary contact and mixing of haplotypes, the deep divergence time implies that the two populations had already been genetically differentiated for millions of years. These findings collectively indicate that the present-day genetic structure of *T. sazae* populations is shaped by both ancient lineage divergence and more recent oceanographic connectivity events.

Yanagimoto et al. [17] further examined *T. sazae* populations along the Japanese coastline and reported genetic structuring influenced by regional ocean currents and historical climatic events. Their findings indicated that populations in the Japan Sea exhibited lower nucleotide diversity (π) compared to those in the Pacific, likely reflecting historical bottlenecks followed by recent demographic expansions. Our results align with this pattern, demonstrating lower π values in East Sea populations (π = 0.003) relative to Jeju populations (π = 0.006). This supports the hypothesis of a recent colonization event in northern waters.

With regard to genetic connectivity between Jeju Island and East Sea populations, the detection of haplotypes associated with both the Tsushima Current (CT) and Kuroshio Current (CK) across these regions provides strong evidence supporting the hypothesis that these ocean currents significantly influence larval dispersal of *T. sazae*. Considering that turban shell larvae exhibit a relatively brief planktonic duration of approximately 3–5 days [16], passive dispersal via ocean currents likely contributes to genetic homogeneity among geographically separated populations.

The relatively low F_ST_ values and AMOVA results indicating that over 100% of genetic variation occurs within populations further suggest minimal genetic structure across the study site, and confirm ongoing gene flow between Jeju and the East Sea, thereby reducing genetic differentiation across these regions. These findings are consistent with previous studies on marine mollusks, which have demonstrated that strong oceanic currents facilitate larval dispersal, maintaining genetic continuity over extensive geographic scales [8,16]. Nevertheless, the observed localized genetic differentiation in certain populations suggests that, despite considerable connectivity mediated by ocean currents, complete homogenization of genetic variation does not occur.

### 4.3. Signals of Potential Localized Genetic Structure

Despite overall genetic connectivity, a few comparisons hinted at a possible localized structure. For instance, the highest F_ST_ values were observed between Shinheung (SH) and Dokdo (DD), as well as Shinheung (SH) and Wangdolcho (WD), albeit without statistical significance. While these values do not provide strong support for distinct population structures, they may suggest weak signals of differentiation that are potentially associated with site-specific factors. The presence of unique haplotypes in certain locations, such as SH and DD, though limited in frequency, may reflect early signs of divergence or localized recruitment.

The unique oceanographic and topographical characteristics of Dokdo and Wangdolcho may facilitate the development of distinct haplotypes and genetic differentiation. Dokdo, formed by volcanic activity 2.5–4.6 million years ago, is geographically isolated and features distinctive seafloor topography [29,30]. The island sits atop a sea-mount rising 2000 m from the seafloor, creating localized current patterns and eddies, potentially trapping larvae and limiting genetic exchange with other populations. The interaction between the Tsushima Warm Current and the North Korean Cold Current near Dokdo creates a dynamic frontal zone [31], which may serve as an oceanographic barrier to larval dispersal, further enhancing genetic isolation.

Similarly, Wangdolcho, located within the Hupo Bank, exhibits complex underwater topography comprising three peaks with the shallowest depth of approximately 6 m [32]. This region is characterized by significant variability in coastal currents, marked by pronounced seasonal fluctuations in current direction and intensity. Additionally, the Wangdol Canyon, descending to depths ranging from 570 to 1600 m, further enhances oceanographic complexity, potentially fostering unique environmental conditions that drive local adaptation and facilitate the emergence of distinct haplotypes [33].

Given the lack of direct empirical studies linking the geographic and oceanographic characteristics of Dokdo and Wangdolcho to genetic isolation in marine invertebrates, the present discussion remains a hypothesis. Future studies should specifically investigate whether the localized current systems, frontal zones, and seafloor structures around these regions indeed promote restricted gene flow and population differentiation. Comparative genetic analyses across multiple marine taxa, combined with detailed oceanographic modeling, would be particularly valuable to validate the role of these unique environmental features in shaping genetic structures in the East Sea.

### 4.4. Climate Change and Habitat Expansion

The haplotype network analysis provides useful insights into the genetic structuring of *T. sazae* populations. The observed star-like topology, centered around haplotype EJ1, indicates populations experiencing recent demographic expansions [34]. This inference is further supported by the relatively higher haplotype diversity coupled with lower nucleotide diversity (π) observed in the East Sea populations, suggesting a relatively recent evolutionary divergence. The presence of multiple East Sea-specific haplotypes that differ by only a few mutational steps from EJ1 reinforces the scenario of recent northward expansion, potentially facilitated by rising seawater temperatures and shifting oceanographic conditions.

Nevertheless, these interpretations must be approached with caution due to the limited number of samples per site (n = 5). Low sample sizes may result in the underestimation of rare haplotypes, incomplete representation of local diversity, and inflated confidence in inferred network topologies, such as the apparent centrality of EJ1. Thus, this study does not provide direct, conclusive evidence of recent range expansion but rather offers preliminary genetic signals that are consistent with a climate-driven northward shift in distribution. These findings should be seen as hypothesis-generating, laying the groundwork for future research with larger sample sizes, multi-locus datasets, and temporal monitoring to confirm whether demographic expansion is indeed ongoing in *T. sazae* populations along the East Sea coast.

Despite these limitations, the findings of this study align closely with previous ecological observations by Son et al. [11], who documented a northward range expansion of *T. sazae* at an average rate of 12.4 km per year from 2009 to 2018, driven primarily by rising seawater temperatures. Their research demonstrated a strong correlation (R^2^ = 0.72) between increasing surface temperatures (0.38 °C per decade) and the species’ expansion rate. The genetic data presented here may further substantiate this ecological pattern, highlighting genetic connectivity between Jeju Island and East Sea populations, a finding consistent with recent climate-driven range expansion.

### 4.5. Implications for Range Expansion and Fisheries Management

The findings of this study provide important insights into the climate-driven range expansion of *T. sazae*. The observed genetic structure indicates that Jeju populations likely serve as a source for newly established populations in the East Sea. This northward expansion appears to be primarily driven by rising seawater temperatures, which have created favorable environmental conditions, promoting the recruitment and persistence of *T. sazae* populations in previously uninhabited regions.

From a fisheries management perspective, these results indicate that *T. sazae* populations in Jeju and the East Sea should be managed as interconnected units rather than isolated stocks. However, the detection of site-specific haplotypes and localized genetic differentiation within certain populations highlights the importance of implementing region-specific management strategies to preserve genetic diversity and ensure the long-term sustainability of the species. Effective conservation measures should incorporate habitat protection, modeling of larval connectivity, and ongoing monitoring of population genetic dynamics in response to continuing environmental changes.

## 5. Conclusions

This study provides preliminary genetic evidence that may support the northward range expansion of *T. sazae*, highlighting the significant role ocean currents play in determining population connectivity. Although populations from Jeju and the East Sea exhibit high genetic connectivity, localized differentiation in certain areas may suggest the influence of region-specific environmental selection pressures. These findings enhance our understanding of marine species’ range shifts, population genetic structure, and their implications for conservation strategies and fisheries management in the context of ongoing climate change. However, as the present study may constrain the resolution of population-level inferences due to the small sample size and the exclusive use of the COI marker, cautious interpretation is required. Future research should aim to incorporate larger sample sizes and utilize multiple genetic markers, including both mitochondrial and nuclear loci, to obtain a more comprehensive understanding of population dynamics as the northward range expansion of *T. sazae* is expected to intensify under climate change.

## Figures and Tables

**Table 1:** Haplotype frequency (%) of *Turbo sazae* populations from Jeju Island and the East Sea. Each column represents a haplotype, and the values indicate the percentage of individuals within each population carrying the corresponding haplotype. Site-specific haplotypes are listed in the rightmost column.

Site	Haplotype Frequency (%)												
	EJ1	E2	E3	E4	E5	E6	E7	E8	J2	J3	J4	Site-specific Haplotypes	
East/Japan Sea	50.0	10.0	10.0	10.0	5.0	5.0	5.0	5.0	-	-	-		
Dokdo (DD)	40.0	20.0	0.0	0.0	20.0	20.0	0.0	0.0	-	-	-	E5, E6	
Pohang (PH)	60.0	20.0	0.0	20.0	0.0	0.0	0.0	0.0	-	-	-	-	
Wangdolcho (WD)	40.0	0.0	20.0	0.0	0.0	0.0	20.0	20.0	-	-	-	E7, E8	
Uljin (UJ)	60.0	0.0	20.0	20.0	0.0	0.0	0.0	0.0	-	-	-	-	
Jeju Island	60.0	-	-	-	-	-	-	-	20.0	10.0	10.0		
Bomok (BM)	40.0	-	-	-	-	-	-	-	20.0	20.0	20.0	J3, J4	
Shinheung (SH)	80.0	-	-	-	-	-	-	-	20.0	-	-	-	

**Table 2:** Summary of genetic diversity indices for *Turbo sazae* populations from Jeju Island and the East Sea. Hd (haplotype diversity) is presented with standard deviation (SD), π denotes nucleotide diversity, and K represents the average number of nucleotide differences within each population.

Site	N	Hd (±SD)	π	K
East/Japan Sea	20	0.747 ± 0.097	0.003	1.663
Dokdo (DD)	5	0.900 ± 0.161	0.003	2.000
Pohang (PH)	5	0.700 ± 0.218	0.002	1.200
Wangdolcho (WD)	5	0.900 ± 0.161	0.005	3.000
Uljin (UJ)	5	0.700 ± 0.218	0.001	0.800
Jeju Island	10	0.644 ± 0.152	0.006	4.111
Bomok (BM)	5	0.900 ± 0.161	0.008	5.300
Shinheung (SH)	5	0.400 ± 0.237	0.006	3.600

**Table 3:** Analysis of molecular variance (AMOVA) results for *Turbo sazae* populations from Jeju Island and the East Sea. The table presents variance components, percentage of variation, and fixation indices (FST) at three hierarchical levels.

Group	Variance Components	Variation (%)	Fixation Indices
Among Regions (East × Jeju)	0.0088	2.44	0.0244
Among populations within regions	−0.0250	−6.97	−0.0714
Within populations	0.3750	104.53	−0.0453

## Data Availability

The data presented in this study are available on request from the corresponding author.

## Supplementary Materials

The following supporting information can be downloaded at: https://www.mdpi.com/article/10.3390/ani15091321/s1, Table S1: Shell height and total weight measurements of *Turbo sazae* individuals collected in the present study.
