# Supplementary material for: Insights into the Genetic Connectivity and Climate-Driven Northward Range Expansion of Turbo sazae (Gastropoda: Turbinidae) Along the Eastern Coast of Korea
Source: Animals (Basel). 2025 May 2;15(9):1321. doi: 10.3390/ani15091321 (PMC12070958; doi:10.3390/ani15091321)
Supplement: Supplementary file 1 [file animals-15-01321-s001.zip › animals-3574471-supplementary.pdf]

animals

MDPI

Table S1. Shell height and total weight measurements of *Turbo sazae* individuals collected in the present study.

Site	Number of Samples	Shell Height (mm)		Total Weight (g)
		Mean ± SD	Min.–Max.	Mean ± SD
Dokdo	5	94.5 ± 4.0	88.3–101.2	193.6 ± 37.7
Pohang	5	71.1 ± 4.2	64.4–78.9	86.8 ± 15.5
Wangdolcho	5	89.0 ± 6.6	78.7–99.4	174.8 ± 46.5
Uljin	5	80.6 ± 5.3	72.5–90.0	112.8 ± 25.7
Bomok	5	75.7 ± 8.6	60.0–88.7	91.0 ± 24.8
Shinheung	5	72.6 ± 4.1	69.0–82.2	83.8 ± 11.5
